# Prevalence of Carpal Tunnel Syndrome Among Computer-Using Non-teaching Staff From Select Educational Institutes at Sangli

**DOI:** 10.7759/cureus.81000

**Published:** 2025-03-22

**Authors:** Swati C Kurane, Sachin Sakate

**Affiliations:** 1 Medical Surgical Department, Bharati Vidyapeeth (Deemed to be University) College of Nursing, Sangli, IND

**Keywords:** carpal tunnel syndrome, mobility disorders, occupational hazard, safe computer use, work-related musculoskeletal disorder

## Abstract

Background

Carpal tunnel syndrome (CTS) is a musculoskeletal condition frequently referred to as an occupational hazard, a classification that includes jobs requiring computer use. Long-term use of computers has been associated with the risk of CTS. Hence, this study aimed to determine the prevalence of CTS among computer-using non-teaching staff from select educational institutes at Sangli, Miraj, Kupwad corporation area.

Methodology

This descriptive, cross-sectional study was conducted from May 2024 to June 2024. A total of 150 computer-using non-teaching staff working at the selected educational institutes of Sangli, Miraj, and Kupwad area were recruited in the study by simple random method. Inclusion criteria included computer-using non-teaching staff of educational institutes who were willing to give informed written consent for the study. Exclusion criteria included participants already on treatment for CTS. A pre-validated structured questionnaire was utilized to assess the sociodemographic variables and signs/symptoms of CTS. The duration and type of computer usage were also recorded.

Results

The prevalence of CTS among computer-using staff was 96.81 (64.54%). Staff aged more than 40 years with a job experience of 10 years were prone to develop CTS. A chi-square value of 51.273 in association with the participant’s age (40 years and more) was observed with a p-value <0.001*, *indicating a significant association of CTS symptoms with the age (40 years and more) of the participants. A significant association of symptoms of CTS with the number of years of computer use (p < 0.001) was also noted. Further, there was a significant association of CTS symptoms with the number of daily working hours using a computer (p = 0.002).

Conclusions

The senior non-teaching staff at the educational institutes were prone to developing CTS due to long-term computer usage. To improve the quality of life of these staff, academic institutions should promote preventive measures for CTS and ensure effective rehabilitative care for those already suffering from CTS. Awareness about the symptoms, causes, and delayed sequel of CTS among regular computer-using staff is a necessary step toward prevention. Regular monitoring of at-risk staff over 40 years of age with long-term usage of computers is necessary for the early detection of CTS. Periodic training about computer-related safe working hours, improved postural habits, and ergonomic workplace practices should be implemented.

## Introduction

Most entrapment neuropathies are caused by carpal tunnel syndrome (CTS), the most prevalent peripheral nerve compression condition in the adult population. CTS primarily affects the median nerve in the wrist and is aggravated by long-term exposure to pressure. The carpal tunnel is a slender space surrounded by bones and ligaments on the palm side of the hand. When the median nerve is compressed, symptoms such as numbness, tingling, and weakness in the thumb and fingers can occur. It is among the musculoskeletal conditions that are referred to as an occupational hazard, including jobs requiring computer use. As per the 2011 census, 9.4% of Indian households own a computer. About 462 million people in the country used the internet in 2016, making up 13.5% of all internet users worldwide. With such a high number of people who use computers and the internet for business and recreation, upper limb nerve injuries such as CTS might become a common condition due to long-term computer usage [1].

Adults with long-term computer usage have an estimated lifetime risk of 10% and an annual incidence of 0.1% for CTS. These estimates were obtained before the significant rise in work-related CTS cases in the 1980s and early 1990s. Considering widespread computerization in the late 1990s, the risk of CTS is much higher in the current scenario. Recent data suggest that 5.8% of women and 0.6% of males in the overall population have CTS. According to data from Sweden, the total prevalence of CTS is 2.1%, as reported by Atroshi and colleagues [2].

The etiology of the CTS is multifactorial. The combination of several risk factors may contribute to the development of CTS. These risk factors can cause chronic irritation and damage to the median nerve [3]. Certain anatomical factors such as inflammatory conditions, neurological conditions, medical diseases, and some occupations are considered prominent risk factors. CTS is a neuropathy caused by the entrapment of the median nerve inside the narrow carpal tunnel, configured by the carpal bones and by the transverse carpal ligament. Compressive forces lead to increased pressure on the median nerve inside the carpal tunnel affecting its function. Clinical features include pain in the hand along with a tingling numbness in the thumb, index, and middle finger. Further progression may cause a reduction in the grip strength and motor function of the affected hand. Symptoms outside the distribution of the median nerve such as elbow, neck, and shoulder pain have also been reported [4].

Patients usually benefit from conservative management. Medications such as corticosteroids, anti-inflammatory drugs, and B complex vitamins have been used. However, CTS prevention is the key to avoiding the progression of motor weakness in the muscles. Prevention strategies such as education in ergonomics, exercise, and physical therapies are recommended to avoid further damage to the median nerve. Alteration in working hours, limiting keyboard usage, and increasing awareness among employees should be encouraged at the institute level [5].

The current study aimed to determine the prevalence of CTS among regular computer-using non-teaching staff at select educational institutes of Sangli, Miraj, and Kupwad corporation area. The study population was involved in long-term computer use, placing them at risk for CTS. The non-teaching staff was not assessed for CTS risk in previous studies; hence, we aim to fill this research gap. 

## Materials and methods

A descriptive, cross-sectional study was conducted at educational institutes. The aim was to determine the prevalence of CTS among regular computer-using non-teaching staff at select educational institutes. Ethical approval was obtained from the Institutional Review Board, Institutional Ethical Committee, Bharati Vidyapeeth Deemed to be University College of Nursing (approval number: EC/NEW/INST/2024/MH/0414). The study was conducted at the select educational institutes of the Sangli-Miraj-Kupwad corporation area. The study duration was four weeks from May 2024 to June 2024.

The study participants were the non-teaching staff from the selected educational institutes of the Sangli-Miraj-Kupwad Corporation area. A total of 150 participants were selected. All participants were regular computer users for their office work. Proper informed and written consent was obtained from all participants. A simple random sampling method was utilized for sample selection. Inclusion criteria included computer-using non-teaching staff of educational institutes who were willing to give informed written consent for the study. The exclusion criteria were participants already on treatment for CTS as the masking of symptoms may affect the data collection.

A pre-validated questionnaire was utilized for data collection. The tool had two sections. Section I contained demographic variables such as age, gender, educational qualification, and duration of computer usage. Section II contained the checklist related to the symptoms of CTS such as tingling, numbness, and pain. The content validity of the tool was established by a committee of experts in the fields of nursing, orthopedics, and statistics. The reliability of the tool was established by the split-half method (r = 0.8). The participants were approached at their respective educational institutes. The data were collected from each participant using the validated tool. SPSS Statistics version 26.0 (IBM Corp., Armonk, NY, USA) was used for data analysis. For the analysis of demographic data, frequencies and percentages were calculated. The association of CTS with select demographic variables such as age and gender was determined using the chi-square test.

## Results

In total, 62 (41%) participants were in the age group of 41-50 years. Overall, 84 (56%) were male participants. Further, 73 (49%) participants were well-educated with postgraduate-level educational status. Overall, 96 (64%) participants were regular users of computers at the office and household for the last 10 year. Moreover, 142 (95%) participants used a computer daily for more than four hours (Table 1).

**Table 1:** Baseline demographic variables of the study participants. n = 150

Demographic variables		Frequency	Percentage (%)
Age in years	20–30	18	13
	31–40	56	37
	41–50	62	41
	51–60	14	9
Gender	Male	84	56
	Female	66	44
Qualification	HSC	3	2
	Diploma	5	3
	Graduate	69	46
	Postgraduate	73	49
Number of years of computer use	Less than 1 year	0	0
	1–5 years	23	15
	6–10 years	31	21
	10 years and above	96	64
Number of daily work hours using a computer	1–3 hours	8	5
	4 hours and above	142	95

Overall, 65-80% of the participants reported symptoms such as tingling, numbness, and pain in their fingers. Moreover, 50-60% of participants reported swelling, stiffness in the fingers, and difficulty in writing. Further, 70-80% of participants reported tingling, reduced sensitivity, and radiating pain in their palms. Overall, 60-70% of the participants reported swelling and stiffness of the wrist affecting the range of motion. In total, 60-80% of participants reported numbness and pain in their elbow. Finally, 70-80% of participants reported pain and numbness in the shoulder along with difficulty during activities (Table 2).

**Table 2:** Symptoms of carpal tunnel syndrome among the study participants. n = 150

Carpal tunnel syndrome symptoms	Yes		No	
	n	%	n	%
Fingers				
Felt numbness	119	79	31	21
Experienced a tingling sensation in the thumb, index, long, or ring fingers	120	80	30	20
Experienced pain in the thumb, index, long, or ring fingers	98	65	52	35
Noticed weakness in grip strength while carrying bags	67	45	83	55
Felt a bump on my fingers	70	47	80	53
Experienced swelling in fingers	78	52	72	48
Experienced difficulty while writing	90	60	60	40
Sometimes felt stiffness with finger movement in one or both hands	90	60	60	40
Palms				
Felt reduced sensitivity in the palms of one or both hands	124	83	26	17
Experienced a tingling sensation in both palms	106	71	44	29
Experienced pain radiating from fingers to wrist	93	62	57	38
Experienced pins and needles sensation in the palms	75	50	75	50
Wrist				
Felt swelling in the wrist of one or both hands	107	72	43	29
Experienced stiffness in the wrist and reduced range of motion in the wrist	93	62	57	38
Experienced pain radiating from the wrist to the elbow	84	56	66	44
Elbow				
Experienced numbness or tingling in the elbow	132	88	18	12
Experienced pain in the elbow	97	65	53	35
Experienced problems with elbow movement in one or both hands	89	59	61	41
Experienced pain radiating from the elbow to the shoulder	60	40	90	60
Shoulder				
Felt numbness when reading a newspaper, steering a car, or knitting	120	80	30	20
Difficulty in performing activities (i.e., driving, riding a motorcycle/bicycle, cutting the grass, using hand tools)	104	69	46	31
Experienced pain traveling to the forearm toward the shoulder	114	76	36	24

The association of CTS symptoms with select demographic variables was determined using the chi-square test. A chi-square value of 51.273 in association with the participant’s age (40 years and more) was observed with a p-value <0.001 (5% level of significance). This indicated that there was a significant association between CTS symptoms and the age (40 years and more) of the participants. A chi-square value of 64.453 in association with the participant’s number of years of computer use was observed with a p-value <0.001 (5% level of significance). This indicated that there was a significant association between CTS symptoms and the number of years of computer use. A chi-square value of 9.832 in association with the participant’s number of daily work hours using a computer was observed with a p-value of 0.002 (5% level of significance). This indicated that there was a significant association between CTS symptoms and the number of daily work hours using a computer (Table 3).

**Table 3:** Association of symptoms of carpal tunnel syndrome with select demographic variables. P-values of 0.05 or less are considered significant. n = 150; *: significant p-values.

Demographic variables	Chi-square value	P-value
Age in years	51.273	<0.001*
Gender	0.15	0.699
Qualification	2.523	0.471
Number of years of computer use	64.453	<0.001*
Number of daily work hours using a computer	9.832	0.002*

## Discussion

CTS is currently regarded as a professional disease based on epidemiological investigations over the past few years. Prolonged keyboard use has been associated with CTS as computer usage has become essential at the workplace. The repetitive and forceful nature of tasks increases the vulnerability to CTS over a long duration. Farmers, assembly line workers, forestry workers, and construction workers are also at risk of developing CTS. Long-term use of computer keyboards and mouse can lead to complex manipulation of small components of the wrist, exerting constant and continuous pressure on the median nerve [6,7]. This study aimed to determine the prevalence of CTS among non-teaching staff of educational institutes using computers for daily office work.

This study observed a high prevalence of CTS symptoms among participants aged 40 years or more. The age-related degenerative changes in the muscle and tendon, loss of myelinated fibers, and demyelination are the suggested pathophysiologic mechanisms for CTS in advanced age [8]. This is in line with previous studies from India, Kuwait, and Pakistan. A study by Alhusain et al (2019) observed that CTS symptoms were more prevalent in those over 41 years of age [9]. However, Abdullatif et al. (2019) in a cross-sectional study observed that CTS was common among the age group of 26-30 years owing to the rising work burden on the practically active population [10].

Gender variance has been noted with CTS. Previous studies suggested that females are at risk for CTS, as they are commonly involved in intricate tasks. The hormonal influence due to pregnancy or contraceptive usage makes them prone to tissue edema, increasing the risk of nerve entrapment. Repetitive movements demanding fine finger manipulation lead to constant pressure on the median nerve in the carpal tunnel. Kurtul and Mazican (2022) observed female predominance of CTS at 73.2% and suggested that it may be due to a smaller carpal tunnel and additional daily work at home which may aggravate the symptoms [11]. Feng et al. (2021) observed similar findings and suggested that it may be related to hormonal and circulatory factors [12]. The risk of CTS significantly rises after menopause due to multiple factors. Al-Rousan et al. (2018) observed a sudden drop in estrogen levels causing changes in forearm fat content leading to CTS and suggested hormonal therapy [13]. In this study, there was no significant association between gender and CTS symptoms.

Both sensory and motor deficits have been noted as a disease spectrum of CTS [14]. Initially, sensory symptoms such as numbness, tingling, and altered sensation in the finger, palm, and wrist are reported. Chronic nerve compression may lead to demyelination, hypoxia, and loss of axons leading to motor symptoms. Thenar muscle atrophy may hamper the ability to perform fine motor tasks. Chronic pain, swelling at the wrist, and fingers progressing to decrease in hand strength and dexterity have been noted in the long term [15,16]. In this study, notable motor symptoms were not observed; however, significant sensory symptoms were experienced by the participants. In this study, 65-80% of the participants reported symptoms such as tingling, numbness, and pain in their fingers. These symptoms were pronounced during working hours and typically improved after rest. This suggests the early stage of the disease. However, Mokhtar (2021) observed tingling, numbness, and pain in the hands which increased at night [17]. This suggests chronic nerve compression and hypoxia.

Participants with longer duration of computer use have high rates of CTS, as suggested in previous studies. Demissie et al. (2021) in a cross-sectional study observed that participants who worked for more than eight hours on a computer were 5.2-fold (adjusted odds ratio (AOR) = 5.2; 95% confidence interval (CI) = 2.8-7.8) more likely to develop CTS and participants with five or more years of work experience were 7.98 times more likely (AOR = 7.98; 95% CI = 3.70-17.33) to develop CTS. This study also suggested that participants with a job duration of more than 10 years had more pronounced CTS symptoms. Moreover, participants using computers for four hours or more had more symptoms of CTS. The risk of the CTS probably increased by repeatedly performing the same tasks in the same working posture for an extended period. Workers performing repetitive movements over time are at risk of CTS. Previous studies confirmed that ergonomics training improves the knowledge of workers and workstation habits, promotes safe working practices, and results in changes in hand/wrist posture when using computers. Significant improvements in symptoms have been noted after ergonomic training for workers. We recommend similar preventive and rehabilitative strategies while working to reduce the severity of symptoms and improve the functional status [18].

The study has achieved its objective of determining the prevalence of CTS; however, it has certain limitations. This study was limited to non-teaching staff of select educational institutes of the Sangli, Miraj, and Kupwad corporation areas, which may not be representative of the entire population. Participants may not accurately report about their computer use or symptoms which could lead to misclassification bias. The sample size was likely too small to detect a significant association between risk factors and CTS. A prospective study on a large population is recommended to establish a causal relationship between computer use and CTS. Exploration of the effectiveness of ergonomic interventions or exercise programs in preventing and managing CTS is needed.

## Conclusions

In this study, the prevalence of CTS was high among the regular computer-using non-teaching staff at the select educational institutes. Age, long duration of employment, and extended daily computer use contributed to the increased risk of CTS. Long-term computer use may increase the risk of CTS, and there is a strong link between computer use and CTS. However, the adverse effects of long-term computer usage need further evaluation by large-scale population-based research. Early detection of CTS symptoms is crucial for the prevention of the adverse effects associated with CTS. Awareness about the symptoms, causes, and delayed sequel of CTS among regular computer-using staff is a necessary step toward prevention. Regular monitoring of at-risk staff over 40 years of age with long-term usage of computers is necessary for the early detection of CTS. Periodic training about computer-related safe working hours, improved postural habits, and ergonomic workplace practices should be implemented.

## Appendices

Study questionnaire

Section I

Instructions

1.     The information will be kept confidential.

2.     Put a tick (√) in front of the correct option.

Code No:

Demographic variables

·       Age in years:

·       Gender:

                  Male:

                  Female:

·       Qualification:

                 HSC:

                 Diploma:

                 Degree:

                 Post-graduation:

·        Use of computer since:

                Less than one year

                1 to 5 years

                5 to 10 years

                10 years and above

·       Use of computer daily

                1-3 hours

                4 hours or more

Section II

**Table 4:** Section 2 of the study questionnaire. CTS = carpal tunnel syndrome

Serial number	Participants’ characteristics of CTS	Yes	No
	Fingers		
1	Felt numbness		
2	Experienced a tingling sensation in the thumb, index, long, or ring fingers		
3	Experienced pain in the thumb, index, long, or ring fingers		
4	Noticed weakness in grip strength while carrying bags		
5	Felt a bump on my fingers		
6	Experienced swelling in fingers		
7	Experienced difficulty while writing		
8	Sometimes felt stiffness with finger movement in one or both hands		
	Palms		
9	Felt reduced sensitivity and numbness in the palms of one or both hands		
10	Experienced a tingling sensation in both palms		
11	Experienced pain radiating from the fingers to the wrist		
12	Experiencing pins and needles sensation in the palms		
	Wrist		
13	Felt swelling in the wrist of one or both hands		
14	Experienced stiffness in the wrist and reduced range of motion in the wrist		
15	Experienced pain radiating from the wrist to the elbow		
	ELBOW		
16	Experienced any numbness or tingling in the elbow		
17	Experienced pain in the elbow		
18	Experienced problems with elbow movement in one or both hands		
19	Experienced pain radiating from the elbow to the shoulder		
	Shoulder		
20	Felt numbness when reading a newspaper, steering a car, or knitting		
21	Difficulty in performing activities (i.e., driving, riding a motorcycle/bicycle, cutting the grass, using hand tools)		
22	Experienced pain traveling to the forearm toward the shoulder		
